# Bilateral Upper Limb Complex Regional Pain Syndrome (Type 2) in Cervical Spinal Cord Injury: A Case Report

**DOI:** 10.7759/cureus.26440

**Published:** 2022-06-29

**Authors:** Soo Ting Kong, Dexter Yeo, Edmund J Neo, Dominic Chen

**Affiliations:** 1 Rehabilitation Medicine, SingHealth Residency, Singapore, SGP; 2 Yong Loo Lin Medical School, National University of Singapore, Singapore, SGP; 3 Rehabilitation Medicine/General Medicine, Sengkang General Hospital, Singapore, SGP

**Keywords:** disability, cervical spinal cord injury, pain disorder, complex regional pain syndrome, physical medicine and rehabilitation

## Abstract

Complex regional pain syndrome (CRPS) is a poorly understood pain disorder presenting with predominantly neuropathic features. It is often, though not always, associated with an injury to the central or peripheral nervous systems, and in such cases may aggravate the prevailing disability. We describe a unique case of bilateral CRPS arising in a patient following traumatic spinal cord injury.

## Introduction

Complex regional pain syndrome (CRPS) manifests as a group of symptoms that include pain, allodynia, swelling, vasomotor impairment, limited range of motion, and motor dysfunction [1]. It can be divided into two subtypes, namely, Type 1, where no nerve damage occurs, and Type 2, where there is associated underlying nerve damage. The literature on bilateral CRPS in central cord syndrome is rare and limited to case reports [2,3].

CRPS is easily overlooked by internists treating pain neuromuscular conditions owing to a heterogeneous range of clinical differentials. The main purpose of this case report is to provide a general overview of CRPS and raise awareness among physicians regarding the diagnostic and treatment complexities when it presents bilaterally.

## Case presentation

A 68-year-old man, with no known drug allergies and a history of ischemic heart disease, for which he had percutaneous coronary intervention in 2003 and was taking aspirin and atorvastatin ever since, hyperlipidemia, and degenerative cervical spondylosis, presented with traumatic central cord syndrome after falling down a flight of stairs.

He underwent a C5-C6 anterior cervical discectomy and fusion and was transferred to the inpatient rehabilitation unit for spinal care. To standardize his neurological assessment, the International Standards for Neurological Classification of Spinal Cord Injury (ISNCSCI) was applied [4]. His motor and sensory examination findings are shown in Table 1. During the initial evaluation, there was no evidence of spasticity.

**Table 1:** Neurological assessment on postoperative day one.

	Motor score (right/left)	Best sensory score (right/left)
C5	5/5	2/2
C6	4/4	2/2
C7	5/5	2/2
C8	1/1	2/2
T1	1/1	2/2
L2	3/2	2/2
L3	4/3	2/2
L4	4/3	2/2
L5	4/3	2/2
S1	4/3	2/2

**Figure 1:**
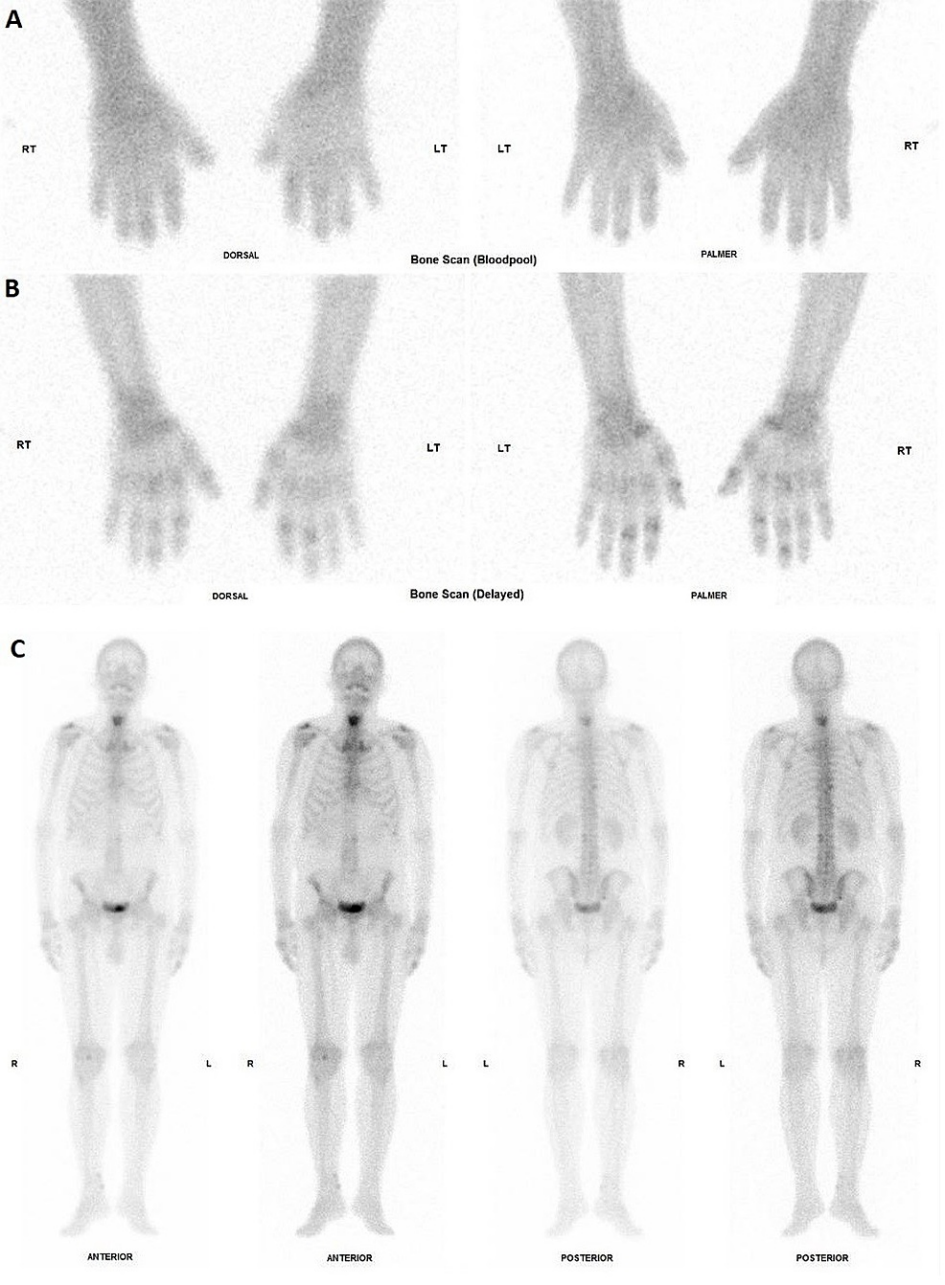
Triphasic bone scan images showing (A) symmetrically increased radiotracer uptake in both hands in the blood pool phase, (B) increased peri-articular radiotracer uptake in the small joints of the hands, and (C) more prominent radiotracer activity in the hands compared to the lower limbs and other major joints.

On the 20th day post-surgery, he developed bilateral hand swelling with pain associated with warmth and redness in both hands, impeding his progress with therapy. He was able to ambulate with contact assistance and perform basic activities of daily living (bADL); however, his fine motor function was affected. He was suspected to have CRPS and was started on prednisolone 30 mg once daily. A triphasic bone scan (TPBS) revealed symmetrical increased radiotracer activity in both hands (Figure 1), confirming the diagnosis.

He underwent three weeks of inpatient rehabilitation. The multidisciplinary team consisted of a rehabilitation physician (physiatrist), a nuclear medicine specialist, occupational therapists (OT), physiotherapists (PT), and a dietitian. The patient received at least five two-hour sessions of PT and OT a week. The sessions emphasized the range of motion, strengthening, endurance, desensitization strategies, and ADL retraining.

His symptoms and functional ability improved after three weeks of steroids and inpatient rehabilitation. For example, his precision grip improved from cylindrical to a tripod when working with clothes pegs and finally to a pincer grip when using the arch ring.

He was discharged to outpatient therapy. However, there was still residual edema noted over his hand, for which he was started on fluidotherapy, acupuncture, compression wrapping, and kinesiology taping. On subsequent outpatient follow-up, he reported that taping was not improving the hand swelling, though the other three physical modalities were effective.

## Discussion

Bilateral Type 1 CRPS (CRPS-1) is often described, though Type 2 CRPS (CRPS-2), in which there is a proven nerve injury, rarely manifests bilaterally, and, even then, has only been reported in the lower extremities. CRPS as a whole may be under-reported as differentials include clinically similar yet more common mimics such as heterotopic ossification (HO) and deep vein thrombosis (DVT) [5-7]. There are no studies on the prevalence of bilateral CRPS in patients with central cord syndrome. Diagnosis of CRPS presently follows the Budapest consensus group’s criteria [Table 2] [1].

**Table 2:** Budapest criteria for the diagnosis of CRPS. CRPS: complex regional pain syndrome

	Criteria	
1	Continuing pain, disproportionate to any inciting event	
2	At least one symptom from at least three of the following categories:	Sensory: reports of hyperesthesia and/or allodynia; Vasomotor: reports of temperature asymmetry and/or skin color changes and/or skin color asymmetry; Sudomotor/edema: reports of edema and/or sweating changes and/or sweating asymmetry; Motor/trophic: reports of decreased range of motion and/or motor dysfunction (weakness, tremor, dystonia) and/or trophic changes (hair, nail, skin)
3	At least one sign from at least two of the following categories:	
4	No other diagnosis that better explains the findings	

Clinical diagnosis is confirmed with the TPBS which is a radionucleotide scan, in which increased radiotracer uptake can be observed during the angiographic, blood pool, and delayed phases of the scan [8]. In our case, the bilateral diagnosis was technically challenging as asymmetrical radiotracer uptake in the affected limb usually assists with the visual diagnosis of a unilateral CRPS, though this was still feasible based on the comparison with other joints in the body.

The treatment of choice for an acute flare of CRPS is corticosteroids. An acceptable starting dose is prednisolone 60 mg with dose-tapering by 5 mg per day until 20 mg, followed by 5 mg per week, although a lower starting dose of 40 mg can be considered to reduce potential side effects in elderly and diabetic patients [9]. We did not make any dose increments to account for the bilateral presentation, and his condition improved on 30 mg of daily prednisolone which we considered a suitable dose for unilateral conditions.

Delay in diagnosis and initiation of treatment during the acute phase is associated with multiple disabling musculoskeletal complications, including muscle atrophy, tendon shortening, contractures, osteoporosis, and arthropathies, which will inevitably disrupt ADL as well as the quality of life [10]. Although our patient experienced a good neurological recovery regarding muscle power, the persistent swelling continued to affect his functional recovery in bimanual tasks.

Promising new treatments for subacute CRPS include other pharmacological options such as ketamine, memantine, intravenous immunoglobulin, and epidural or intrathecal medications such as baclofen and clonidine [10]. The role of sympathetic blockade remains under scrutiny. Beyond these, rehabilitation goals should remain focused on pain relief, functional restoration, and psychological stabilization of the patient. Conventional physical and occupational therapy are augmented by physical modalities such as kinesiology tape, electrical stimulation, and mirror therapy. Fluidotherapy is a form of dry heat therapy involving heat transfer through fine particles circulating within a chamber of heated air that can offer both superficial heat and tactile stimulation. It has been utilized to alleviate musculoskeletal pain and swelling. The addition of fluidotherapy on top of conventional rehabilitation has also been reported to reduce neuropathic pain and edema in CRPS in a post-stroke population [11].

## Conclusions

We report a rare case of bilateral CRPS following central cord syndrome and describe the diagnosis and multimodal management of this challenging condition. We look forward to future studies to shed light on new strategies in the assessment and management of such patients. When in doubt, referral to rehabilitation medicine can aid in prompt diagnosis and early treatment to address pain and functional impairments.
